# MET‐FTD: Music‐based Empathy Training for Frontotemporal Dementia

**DOI:** 10.1002/alz70858_106125

**Published:** 2025-12-26

**Authors:** Kaitlin Seibert, Elena Badillo‐Goicoechea, Grace Lee, Miles Breese, Mia Krings, Rebecca J.F. Freiman, Jeffrey Wolfe, Borna Bonakdarpour

**Affiliations:** ^1^ University of Chicago, Chicago, IL, USA; ^2^ Northwestern University, Chicago, IL, USA; ^3^ Healthy Aging and Alzheimer's Research Care Center, University of Chicago, Chicago, IL, USA; ^4^ Greater Chicago Music Therapy Inc, Chicago, IL, USA; ^5^ Institute for Therapy through the Arts, Evanston, IL, USA; ^6^ Mesulam Center for Cognitive Neurology and Alzheimer's Disease, Chicago, IL, USA

## Abstract

**Background:**

Behavioral variant frontotemporal dementia (bvFTD) often presents with social cognition and behavior changes out of proportion to cognitive decline. BvFTD has the highest caregiver burden of any dementia, leading to significant marital problems not infrequently ending in divorce. One factor in such social cognition deficits is lack of empathy, a core diagnostic feature in bvFTD. Empathy is defined as the ability to recognize and/or adopt another person's emotional state while maintaining self‐other distinction. While several interventions have attempted to target lack of empathy, none have adequately alleviated the emotional burden of families with bvFTD. Based on the neuroanatomical overlap of social cognition, music, and atrophy in bvFTD and inspired by music therapy interventions for social cognition disorders such as schizophrenia and autism, we created a 5‐stage music‐based empathy intervention protocol focused on improving empathy in bvFTD: Music‐based Empathy Training for Frontotemporal Dementia (MET‐FTD).

**Method:**

In this preliminary feasibility study, we implemented a pilot group to determine the feasibility of a 12‐week at‐home empathy‐based music intervention, MET‐FTD, in patients with bvFTD. Participants were enrolled by their providers from University of Chicago Memory Center and Northwestern University Mesulam Center. The MET‐FTD curriculum was administered by licensed music therapists at Institute for Therapy through the Arts and Greater Chicago Music Therapy Inc. Advancement by stage across a total of 7 stages was determined by a consensus group based on success rate of socioemotional cue opportunities. Empathy was measured before and after the intervention by Interpersonal Reactivity Index (IRI).

**Result:**

Five individuals with bvFTD were enrolled and four participants successfully completed the 12‐week MET‐FTD curriculum. One participant, the most advanced in disease and a musician, remained in Stage 1 throughout the intervention. One participant gradually moved through the curriculum and ended at Stage 4 and two participants reached Stage 5. Preliminary IRI data (*N* = 4) showed a mean improvement in IRI score of 6 after the intervention compared to before the intervention.

**Conclusion:**

MET‐FTD is feasible in individuals with bvFTD and may improve empathy. Larger, higher‐powered studies are needed to determine the efficacy of music therapy interventions for empathy in bvFTD.

